# Computed tomography in the diagnosis of intraperitoneal effusions: The role of texture analysis

**DOI:** 10.17305/bjbms.2020.5048

**Published:** 2021-08

**Authors:** Csaba Csutak, Paul-Andrei Ştefan, Roxana-Adelina Lupean, Lavinia Manuela Lenghel, Carmen Mihaela Mihu, Andrei Lebovici

**Affiliations:** 1 Radiology and Imaging Department, County Emergency Hospital, Cluj-Napoca, Cluj, Romania; 2 Radiology, Surgical Specialties Department, “Iuliu Hațieganu” University of Medicine and Pharmacy, Cluj-Napoca, Cluj, Romania; 3 Anatomy and Embryology, Morphological Sciences Department, “Iuliu Hațieganu” University of Medicine and Pharmacy, Cluj-Napoca, Cluj, Romania; 4 ”Dominic Stanca” Obstetrics and Gynecology Clinic, County Emergency Hospital, Cluj-Napoca, Cluj, Romania; 5 Histology, Morphological Sciences Department, “Iuliu Hațieganu” University of Medicine and Pharmacy, Cluj-Napoca, Cluj, Romania

**Keywords:** Ascites, computed tomography, CT, diagnostic methods, image processing, texture-based analysis, peritoneal neoplasms

## Abstract

The morphological changes suggesting peritoneal carcinomatosis are inconsistent and may be visible only at later stages of the disease. However, malignant ascites represents an early sign, and this fluid exhibits specific histological characteristics. This study aimed to quantify the fluid properties on computed tomography (CT) images of intraperitoneal effusions through texture analysis and evaluate its utility in differentiating benign from malignant collections. Fifty-two patients with histologically proven benign (n=29) and malignant (n=23) intraperitoneal effusions who underwent CT examinations were retrospectively included. Texture analysis of the fluid component was performed on the non-enhanced phase of each examination using dedicated software. Fisher and the probability of classification error and average correlation coefficients were used to select two sets of ten texture features, whose ability to distinguish between the two types of collections were tested using a k-nearest-neighbor classifier. Also, each of the selected feature’s diagnostic power was assessed using univariate and receiver operating characteristics analysis based on the calculation of the area under the curve. The k-nearest-neighbor classifier was able to distinguish between the two entities with 71.15% accuracy, 73.91% sensitivity, and 68.97% specificity. The highest-ranked texture parameter was Inverse Difference Moment (*p*=0.0023; area under the curve=0.748), based on which malignant collections could be diagnosed with 95.65% sensitivity and 44.83% specificity. Although successful, the texture assessment of benign and malignant collections is less effective in reflecting the cytological differences between the two groups.

## INTRODUCTION

Peritoneal carcinomatosis (PC) is defined by the intraperitoneal dissemination of any tumor which does not originate from the peritoneum itself [1]. The imaging findings in PC can range from simple fluid accumulation to nodules and infiltrative masses [2]. Although most morphological changes indicating PC are inconsistent and may be visible only at later stages of the disease [3], malignant ascites represents an early manifestation that may be encountered in most patients with PC [2].

Common imaging methods in the PC evaluation are represented by ultrasonography (US) and computed tomography (CT) [4]. US can identify free intra-abdominal collections with over 90% sensitivity and specificity [5], while also being able to demonstrate internal debris and septa more accurately than CT [6]. However, centrally located inoculations (e.g. in the mesentery) cannot be highlighted by this method because of the acoustic impedances created by intestinal gas and abdominal adipose tissue [7, 8]. Overall, contrast-enhanced CT examination provides a sensitivity of 25-100% and a specificity of 78-100% in the preoperative staging of PC [9, 10]. But such high diagnostic power is often achieved after visualization of advanced changes of PC (greater and lesser sac ascites, omental cake sign, etc.), together with the overall assessment of abdominal organs [11]. When peritoneal implants are the only visible manifestation of PC, the CT ability to identify PC decreases to 57.14% sensitivity almost null specificity [3, 11]. However, both techniques mostly rely on identifying the morphological changes suggestive of PC, and for this reason, their contribution in diagnosing PC without a circumscribed tumor and in differentiating peritoneal implants from scar tissues is limited [12].

The CT examination can detect intra-peritoneal effusions (IPEs) as small as 50 ml [13]. Apart from being an early sign of PC [2], the pathological analysis shows that this type of intra-peritoneal effusion (IPE) has several particularities in terms of biochemical, cytological, and physical features [14,15]. It is desirable that these distinctive features be also reflected on CT images, and could carry additional diagnostic information, but they are difficult to quantify during the routine evaluation of medical images.

Textures represent patterns of shapes and colors formed by the pixels within a digital image [16]. Texture analysis (TA) is a technique based on the extraction and processing of image-specific parameters, being able to provide an objective description of image contents by quantifying the distribution patterns and intensity of the pixels [17]. By specific parameters, TA can offer information about the tissue characteristics, and its utility has been proven especially in the diagnosis and prognosis of oncological pathologies [18].

In the present study, TA was used to quantify the image features of IPEs on CT images. The aim was to determine if texture parameters could provide additional diagnostic information to be used as a non-invasive criterion for distinguishing between benign and malignant IPEs.

## MATERIALS AND METHODS

### Patients

This Health Insurance Portability and Accountability Act–compliant, single-institution study was approved by the institutional review board (Ethics Committee of the “Iuliu Hațieganu” University of Medicine and Pharmacy Cluj-Napoca; registration number, 50/11.03.19), and a waiver of informed consent was obtained owing to its retrospective nature. In our radiology information system, reports of abdomino-pelvic CT scans were searched for the period May 2019 - January 2020 using the keywords:” ascites”, “intra-abdominal collection/s” and “peritoneal carcinomatosis”. The original search yielded 238 reports. Each report was then analyzed and the studies which did not report the presence of ascites were excluded (n=29). The remaining 209 studies were reviewed by one researcher to confirm the existence of the intra-abdominal fluid collections. The patients’ records were retrieved from the archive of our healthcare unit and investigated for disease-related data. The inclusion criteria were: a minimum transversal diameter of the collections of at least 30 mm, the existence of a final pathological diagnosis of the fluid, the absence of multiple synchronous pathologies that could have cause ascites, the absence of imaging artifacts, and the pathological analysis of the fluid being performed at less than 20 days before or after the CT examination. After applying these criteria, 52 patient records were included in this study.

### Reference standard

Eight patients had a final diagnosis of cirrhosis without imaging evidence of hepatocellular carcinoma. Cardiac ascites was found in two patients with cardiac failure (in both cases, no associated pathologies that could have caused intraperitoneal fluid accumulation were detected). Intra-abdominal abscesses (or secondary peritonitis) were due to cholecystitis in two patients, appendicitis in three, and a postoperative abscess in one subject. Three patients had intraperitoneal collections from acute perforations of the upper gastrointestinal tract by benign ulcers. Pancreatitis-related collections were due to pancreatic ascites in four patients, infected peripancreatic collections in two, and one subject with pseudocysts.

Twenty-three patients suffered from oncological disease. Regarding ovarian cancers, nine patients were diagnosed with serous carcinoma, three with clear cell carcinoma, one with malignant mixed Müllerian tumor, and one with transitional cell carcinoma.

Laparoscopy was performed in 12 subjects, paracentesis in 13, and laparotomy in 27 patients. The same laboratory analyzed all the fluid samples. Each sample underwent cytological and biochemical analysis, as well as additional ancillary tests. The cytological analysis included a first step where the probes were centrifuged. Secondly, two pellets were then assembled from each probe, stained with hematoxylin and eosin and submitted to microscopical analysis. Tumoral cellularity was detected in the peritoneal fluid of 23 patients, which were included in the malignant IPEs group. The rest of the subjects were included in the benign IPEs group (Table 1).

**TABLE 1:**
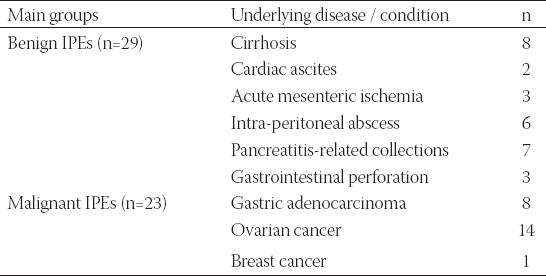
Patients

### CT protocol

All CT scans were performed on the same unit, Siemens Somatom Sensation, 16 slices (Siemens medical solutions, Forchheim, Germany). The CT scan covered the region from the dome of the liver to the ischial tuberosity attachment. The parameters of the CT scan were 120 kV, 200 mAs, slice thickness of 3 mm.

### Texture analysis and fluid classification

The radiomics approach consists of four steps: image segmentation using regions of interest, feature extraction, feature selection, and prediction. Each examination was reviewed on a dedicated workstation (General Electric, Advantage workstation, 4.7 edition) by two radiologists (C.C. and, A.L. each with at least 15 years’ experience in abdominal imaging), who were also blinded to the clinical picture. On the non-enhanced phase of each examination, the two researchers developed a common opinion about choosing a slice they considered the most representative for the fluid content. All examinations were anonymized, and the selected slices were retrieved in DICOM format (Digital Imaging and Communications in Medicine). A third researcher (P.A.S.) imported each image into a texture analysis software, MaZda version 5 [19].

### Image segmentation

**FIGURE 1:**
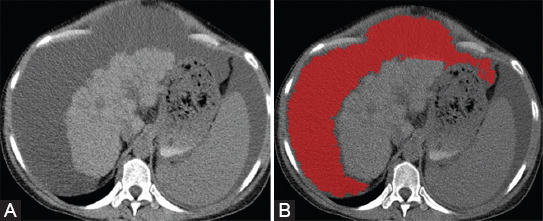
(A) Axial CT non-enhanced phase image of a 58-year-old patient with cirrhosis. (B) The slice with the region of interest (red area) used for texture analysis.

For the segmentation step, the same researcher (P.A.S.) incorporated the ascitic fluid in a two-dimensional (2D) region of interest (ROI). A semi-automatic level-set technique was used for the definition and positioning of each ROI. The researcher placed a seed in the approximate center of the fluid collection and the software automatically delineated the collection based on gradient coordinates (Figure 1). A limitation of dynamics to μ±3σ (μ=gray-level mean; and σ=gray-level standard deviation) was applied to reduce the influence of contrast and brightness that could affect the true image textures [20].

### Feature extraction

The feature extraction was automatically performed by the built-in tools of the MaZda software. The analysis of every ROI resulted in over 300 texture parameters which originated from the grey-level histogram, the wavelet transformation, the co-occurrence matrix, the run-length matrix, the absolute gradient, and the autoregressive model.

### Feature selection

The MaZda program allows the selection of the most discriminative features through several reduction techniques. One such technique is represented by the Fisher method. The Fisher coefficient (F) defines the ratio of between-class variances to within-class variances. This method provides a set of ten features that have a high discriminatory ability [20]. Alongside the Fisher method, another selection technique based on the probability of classification error and average correlation coefficients (POE + ACC) was utilized [21]. By applying these selection methods, two sets, each containing ten features, were selected. The two selection techniques were associated in previous studies [22,23], showing good classification results.

### Feature prediction

In the prediction step, two methods were used to evaluate textural parameters’ ability to distinguish between the two IPEs groups. Firstly, the two sets of parameters selected by Fisher and POE+ACC methods were imported in into the B11 program [19]. Within the B11 program, the capacity of feature sets to differentiate between various classes can be further explored, using classifiers. The classifier used in this step was the k-nearest-neighbor (k-NN) (which follows the partitioning method for clustering) [24]. The k-NN’s ability to distinguish between the two types of effusions was shown by quantifying its accuracy (expressed as a percentage of correctly classified lesions), sensitivity (true positive rate) and specificity (true negative rate). Secondly, the absolute values recorded by the two types of fluids for each parameter were compared using a univariate analysis test (Mann–Whitney U). The receiver operating characteristic (ROC) analysis was performed, with the calculation of the area under the curve (AUC) with 95% confidence intervals (CIs) for the parameters showing P values below 0.0025 (after Bonferroni correction) on the univariate analysis. Statistical analysis was performed using a commercially available dedicated software, MedCalc version 14.8.1 (MedCalc Software, Mariakerke, Belgium).

## RESULTS

Of the 238 patients that referred to our department during the study period, 52 were retrospectively included in our study (24 females, 28 males; mean age, 62.13 years, age range 34-87 years). Subjects were divided according to the final cytological results of their sampled fluid into benign (n=29) and malignant IPEs (n=23). The mean time between the CT examination and the fluid sampling was 9.7 days (range, 2-19 days).

**FIGURE 2:**
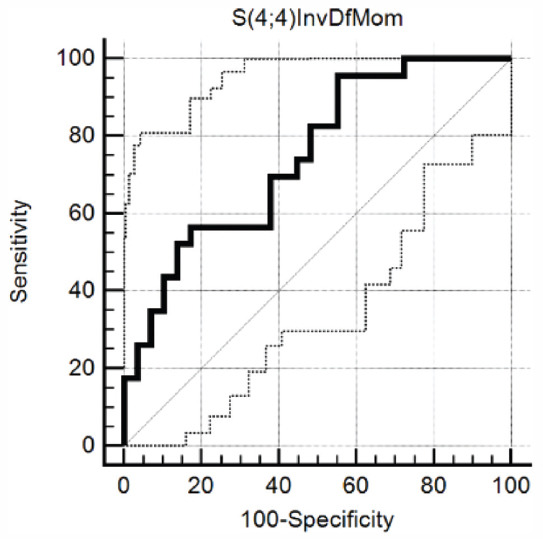
The receiver operating characteristics curve of the S(4,4)InvDfMom (Inverse Difference Moment) parameter for distinguishing malignant from benign ascites.

The sets of features highlighted by each of the selection methods are displayed in Table 2. The S(4,4)InvDfMom (inverse difference moment) parameter was selected by both methods, in each case yielding the best classification potential (as having the highest Fisher and the lowest POE+ACC coefficients). The same parameter was the only one that showed statistically significant results when comparing the absolute values of the parameters contained in each feature set (*p*=0.0023). The average values recorded for S(4,4)InvDfMom were 0.079 [IQR (Interquartile range), 0.076 - 0.083] for benign and 0.084 (IQR, 0.08 - 0.085) for malignant collections. The ROC analysis showed that this parameter was able to distinguish malignant from benign IPEs with a sensitivity of 95.65% (CI, 78.1 - 99.9%) and a specificity of 44.83% (CI, 26.4 - 64.3%) for a cut-off value of > 0.0787, a significance level of 0.0002 and an AUC of 0.748 (CI, 0.608 - 0.858) (Figure 2).

**TABLE 2:**
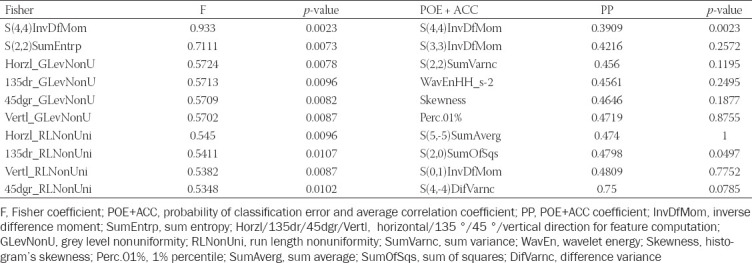
The sets of parameters highlighted by the selection methods and the univariate analysis results (*p*-values).

The k-NN’s performance in distinguishing the two groups is shown in Table 3. Two patients with cirrhosis, two with secondary peritonitis, one with ulcer perforation, two with gastric cancers, two with serous ovarian carcinomas, and one subject with transitional cell carcinoma were misclassified following the processing of features selected by both Fisher and POE+ACC method. Overall, the best performance of the k-NN classifier was achieved following the computation of POE+ACC selected features (accuracy= 71.15%, sensitivity= 73.91%, and specificity= 68.97%).

**TABLE 3:**
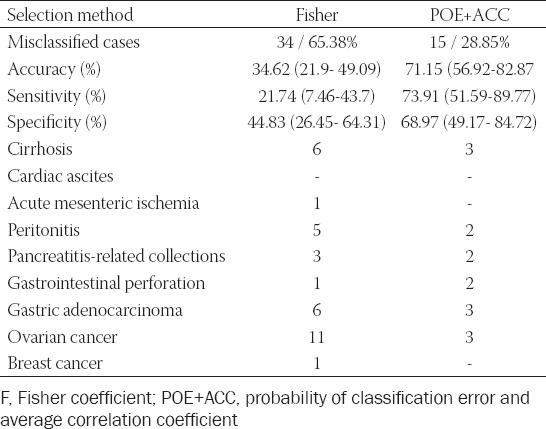
The performance of the k-nearest-neighbor classifier in distinguishing between the two groups, and the numbers of misclassified samples from each histopathological entity. Between the brackets are values corresponding to the 95% confidence interval

## DISCUSSION

Our results indicate that the S(4,4)InvDfMom was the only parameter that showed statistically significant results at the univariate analysis (p=0.0023). Based on the values recorded by this parameter, the distinction of malignant from benign collections showed a remarkably high sensitivity (95.65%) that unfortunately came at the cost of a relatively low specificity (44.83%). InvDfMom reflects the local homogeneity of an image. The value of this parameter rises when more pixel pairs are close to gray-scale value, resulting in higher values for homogeneous images [25]. We recorded higher values of this feature for the malignant than for the benign group.

It was expected that the malignant group would show a higher degree of heterogeneity, this feature being a known characteristic of malignant tissues [26]. On the other hand, benign IPEs can express various types of appearances, mostly depending on the underlying pathology [27,28]. For example, the gross evaluation can identify clear fluid (mostly in liver cirrhosis), or the accumulations can have a cloudy appearance (in pancreatitis, intestinal perforations and bacterial peritonitis) [27]. Our benign IPEs group included multiple types of collections (free fluid, incapsulated, inflammatory and purulent), thus expressing broad density variations on CT images. This density variations were quantified by the TA software in the form of the InvDfMom parameter, which consecutively increased its values in the benign group.

**FIGURE 3:**
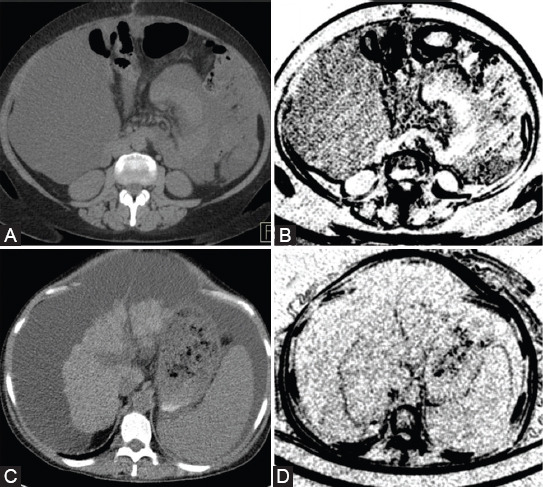
Generated texture maps showing differences between benign and malignant intra-peritoneal collections; (A) a CT image of a 68-year old patient with histologically-proven malignant ascites and (B) generated map based on the Inverse Difference Moment texture feature extracted from Figure 4.A; (C) a CT image of a 58-year old patient with cirrhosis; and (D) generated map based on the Inverse Difference Moment texture feature extracted from Figure 4.C.

Although including only unorganized collections in the benign group would have led to different results, this would have caused a potential bias in our study. However, certain macroscopic differences can be observed based on the maps that show the distribution of the S(4,4)InvDfMom parameter over the two types of effusions (Figure 3). Considering these observations, the texture analysis of the two groups most likely highlighted the differences of the fluids physical properties rather than the histopathological group to which the collections belonged.

Another aspect that calls into question the TA results is the lack of presence of the first order histogram parameters in the sets selected by Fisher and POE+ACC. The histogram analysis reflects only the pixel intensity values, and not the spatial relations between the pixels [29]. It would have been expected that the malignant collections would express higher densities on the CT images, due to their dense cellular population [30] and frequent blood contamination [11], and therefore higher values of the histogram parameters. The first order parameters could not be used for differentiating between the two types of collections for two reasons. Firstly, malignant IPEs often contain chylomicrons, being one of the most common causes of chylous ascites [28].The presence of fat components would automatically decrease the densities recorded on CT images and therefore the values of the histogram parameters. Secondly, several collections included in the benign group are characterized by increased viscosity and high cellularity [31], especially in the case of infectious and inflammatory accumulations, which consequently increased the density and histogram parameters values recorded in these types of IPEs, thus reducing the differences between the two groups.

Similar research that analyzed the capability of texture features to discriminate between benign and malignant ascites based on CT images was conducted by Baroud et al [32]. Besides using the same TA software (MaZda), the workflow was almost entirely different: the feature reduction techniques were based on Fisher and Mutual Information, and the feature vectors were classified using the linear discriminant analysis method. Following this approach, the researchers were able to distinguish collections associated with peritoneal carcinomatosis from ascites without underlying malignancy in 86.5% of cases, and from peritonitis-related ascites in 76.2% of images. Although the percentages of correctly classified images were similar to our results, the authors concluded that TA seemed feasible in distinguishing different types of ascites and could increase the diagnostic confidence in differentiating between different types of ascites [32]. In a previous article [33] we demonstrated that the texture features of ascites extracted from magnetic resonance images showed good capability in differentiating benign from malignant collections. In the above-mentioned research [33], after applying the same two selection methods, the best results were obtained by an artificial neural network classifier after importing the Fisher selected features (accuracy 91.84%; sensitivity 100% and specificity 42.86%). However, based on magnetic resonance (MRI) images, two different texture parameters showed adequate discriminative power (Run Length Nonuniformity computed from vertical and horizontal directions) [33].

Positive cytology can diagnose malignant ascites with almost 100% specificity but with variable sensitivity rates [34]. By using a highly elaborated protocol, Runyon et al. [35] were able to identify malignant cells in 96.7% of the patients with peritoneal carcinomatosis. However, it is safe to assume that not all healthcare units follow such a rigorous workflow process as the one described in the abovementioned study, which included a single method of fluid sampling (paracentesis), a reevaluation of the initial negative results with a second sampling of a larger specimen, and strict coordination between the timing of the sampling procedure with the laboratory [35]. Firstly, our workflow model included several sampling methods, no strict coordination between the laboratory and the sampling procedure, and no reevaluation of the initial negative results during the same hospitalization period. Secondly, we were unable to retrieve any information about the state of the fluids and the transportation procedures that could also alter the quality of this analysis [35].

Our study has several limitations. First, due to its retrospective design, it may have selection and verification bias. Also, being a pilot study, our research incorporated a relatively small number of patients. Second, the only criterion that decided the subjects’ inclusion in each histopathological group was the cytological analysis (the presence of the absence of malignant cells in the fluid samples), which implies several pitfalls that we previously addressed. Third, the ROI segmentation employed in this pilot study comprised a single largest cross-section-based delineation instead of a multi-slice or three-dimensional volume analysis. Also, the inter- and intra-observer agreement was not assessed. Also, the MaZda software used in this article could be considered as outdated, as there were no official updates for in several years. However, in this study, we used a newly developed Beta version of this software, released in 2016 (available at https://data.mendeley.com/datasets/dkxyrzwpzs/1). Although more modern dedicated TA software packages are available, MaZda steel represents a popular TA method, since it provides one of the largest numbers of feature customization, selection, extraction and processing methods. Also, it offers an intuitive interface, and thus the possibility of being used by non-image processing specialists, such as regular physicians.

## CONCLUSION

In conclusion, our objective, namely to assess ascites fluid with texture analysis in order to determine non-invasive differentiation criteria for benign and malignant IPEs, showed statistically significant results. However, it is not clear whether these differences were determined by the malignant cellularity or by other cytological, biochemical or physical fluid properties.
